# Development of key performance indicators for a telemedicine setting in Egypt using an electronic modified Delphi approach

**DOI:** 10.1186/s12913-025-12733-6

**Published:** 2025-07-01

**Authors:** Sara Ebraheem, Ayat Farouk Manzour, Ahmed Elbokl, Tamer Emara, Moustafa El Houssinie, Mahi Mahmoud Al-Tehewy

**Affiliations:** 1 Department of Healthcare Quality, Faculty of Medicine, Ain Shams University, Cairo, Egypt; 2 Department of Community, Environmental and Occupational Medicine, Faculty of Medicine, Ain Shams University, Cairo, Egypt; 3 Department of Neurology, Faculty of Medicine, Ain Shams University, Cairo, Egypt; 4 Ain Shams University Virtual Hospital, Cairo, Egypt; 5 The General Authority for Healthcare Accreditation & Regulation, Cairo, Egypt

**Keywords:** Quality indicators, Key performance indicators, Telemedicine, Delphi method

## Abstract

**Background:**

Performance measurement is crucial for maintaining quality of care in health systems, and so telemedicine is no exception. Key Performance Indicators (KPIs) are some of the primary tools for performance measurement. While there are many global studies on telemedicine quality, studies that are done specifically on indicators are limited. Based on this, the purpose of this study is to develop KPIs for evaluating the performance of telemedicine settings.

**Methods:**

A consensus-based methodological study employing the Delphi method was conducted. The study was conducted in Ain Shams Virtual Hospital (AVH). It started with defining the selection criteria to be used in Delphi method. Then a review of scientific sources was carried out to enlist the potential indicators of telemedicine. An electronic modified Delphi technique was then used. The panel members were invited to rate the indicators on a 10-point Likert scale, and subsequently, the experts then reconsidered their previous voting in the light of the aggregated results. The agreement was reached as the indicator showed a 70% level of agreement. Cohen Kappa (K) was calculated to show the percentage of agreement between the two rounds.

**Results:**

The multi-voting process established three selection criteria for indicators: clarity, feasibility, and importance. A review of research sources led to an initial list of 52 potential indicators, categorized into operations, clinical services, and customer satisfaction. The Delphi method was carried out in two rounds, with expert participation rates of 35% in the first round, and 80% in the second. In the end, 31 indicators achieved the 70% agreement level, with the majority falling under the operations category, while all customer satisfaction indicators were selected. Cohen’s Kappa analysis showed that nearly 85% of comparisons between the two rounds demonstrated substantial agreement.

**Conclusions:**

This study identifies a thorough collection of indicators that offers a valuable tool for enhancing quality in telemedicine settings. The indicators cover important domains in telemedicine services, such as: access, utilization, system efficiency, and customer satisfaction. Future directions should include piloting these KPIs in telemedicine systems and assessing the acceptability of their application.

**Supplementary Information:**

The online version contains supplementary material available at 10.1186/s12913-025-12733-6.

## Introduction

The healthcare industry considers telemedicine a crucial sector. Due to swift advancements in information and communication technologies, telemedicine has expanded its reach significantly [1]. Accordingly, integrating telemedicine and virtual care into the healthcare system enhances efficiency in delivering medical services [2]. This approach aids medical centers in handling prolonged wait times and mitigating the risk of disease progression [3].

The demand for telemedicine surged during pandemics, such as the COVID-19 pandemic [4]. At one point, hospitals were overwhelmed, failing to accommodate all patients and placing significant strain on the healthcare system [5]. This highlighted the urgent need to expand virtual hospitals, which would require a well-designed and effectively implemented system [6]. Generally, virtual hospitals would provide various services, including outpatient clinics, chronic disease management, and video consultations between healthcare providers [7].

Egypt’s pursuit of Universal Health Coverage has created a growing demand for telehealth services; however, progress in their implementation remains limited, emphasizing the need for better preparedness in this area [8]. To elaborate further, the rapid transition to telehealth during the COVID-19 pandemic occurred without sufficient planning, highlighting the importance of further research into public experiences. These experiences would have telehealth adoption and sustainability both during and after the crisis [9].

There is a vital need for assessing and monitoring the performance of health services to ensure high-quality care [10, 11]. As telemedicine has gained traction among consumers, providers, payers, and various stakeholders within the healthcare system, it has become increasingly imperative to evaluate its performance [12]. While comprehensive performance measurement systems are essential, many hospitals prioritize legal compliance and accreditation standards, resulting in limited evaluations that fail to capture the full scope of performance [13]. For meaningful improvement, it is crucial to incorporate diverse input, process, and outcome measures [14]. Common methods traditionally used include: regulatory inspection, public satisfaction surveys, audits, third-party assessments, and statistical indicators [14, 15].

Key Performance Indicators (KPIs) stand out as some of the primary tools for performance measurement [16]. These indicators are viewed as decision-making tools based on performance, and are utilized by policymakers and managers [10, 17]. Generally, indicators usually provide a quantitative measure essential for evaluating, controlling, and monitoring a hospital’s present status [16, 18, 19]. To evaluate the success of implementing telemedicine services, specific and appropriate key performance indicators are required. Furthermore, any accreditation standards of the hospitals require the existence of a set of KPIs to measure the hospital performance [20].

While there are many studies on telehealth quality globally, research done on indicators specifically is limited. Most of these studies have focused on patient satisfaction as an indicator, highlighting factors like improved outcomes, ease of use, and cost-effectiveness [21–24]. As far as is known, there have been no studies conducted to measure performance in a telemedicine by a set of indicators. Consequently, the proposed study represents a cornerstone for such research endeavors in the region to bridge this gap by proposing a comprehensive set of KPIs. Based on this, the main objective of the current study is to identify, adapt, and adopt a set of Key Performance Indicators (KPIs) for monitoring performance in a telemedicine setting in Egypt. These KPIs would be a good reference for any other telemedicine settings especially in low- and middle-income countries. Furthermore, these indicators would support data-driven decision-making, enhance accountability, and ensure telemedicine services are equitable, of high-quality, and cost-effective to meet the needs of all the stakeholders.

## Methods

### Study design

This study is typically categorized under health services research or performance measurement development. A consensus-based methodological study employing the Delphi method was conducted. Specifically, the study utilizes an electronic modified Delphi technique to develop Key Performance Indicators (KPIs) for evaluating the telemedicine settings. The study integrates qualitative insights from expert opinions with quantitative metrics (e.g., Likert scale ratings, agreement percentages, and statistical measures) to achieve its objectives, making it inherently mixed methods that utilize the strengths of both approaches.

The study was conducted in three primary phases: defining the selection criteria, identifying the potential indicators, and conducting the modified Delphi method (which is the main approach in this study). The Delphi method serves as both a forecasting technique and a structured communication approach, involving iterative rounds of questionnaires circulated to a panel of experts [25].

### Study setting

The study was conducted in Ain Shams Virtual Hospital (AVH). The hospital provided telemedicine services through video-consultations for patients in Egypt and some African countries. AVH is a telemedicine service provider operating under a “Treat and Teach” initiative. This initiative was launched in 2016 at the League of Arab States, with the participation of 13 Arab and African countries, along with international telemedicine experts, healthcare providers, and policymakers. AVH also offers teleconsultations through its website, where patients use a customized booking system to provide a brief medical history, select a specialty, or request assistance in choosing the right consultant. Patients then meet with their consultants via video consultation at the scheduled time and are able to upload lab results, images, or other relevant medical documents [26].

### Phase 1, defining selection criteria

A review of studies was performed to identify some criteria for selecting the indicator to be used in the Delphi. A long list containing any criteria that were previously used in the indictor-selection research was developed. Along with two experts, these criteria were refined to develop a shortlist based on the following: redundancy elimination, avoiding duplication, and removing synonymous (same meaning) criteria. 10 experts in quality, public health, and telemedicine were invited to voluntarily participate in a multi-voting process. They were asked to select and rank 5 criteria, assigning each a priority from 5 to 1. The data were analyzed to reveal the highest 3 criteria.

### Phase 2, identifying potential list of indicators

The next phase involved was done by using various sources to identify the indicators to be used in evaluating the performance of telemedicine. Initially, existing and potential quality indicators were determined through a review of international scientific literature. The search was conducted through search engines as PubMed. The search was limited to studies published in English and included the accessible studies published till October 2022. The keywords used for searching were a combination of “telemedicine”, “performance evaluation”, “quality indicators”, and “quality measures”. The Boolean OR AND operators were placed between keywords in the searches.

In addition, grey literature was explored, concentrating on international organizations involved in telemedicine, including the American Telemedicine Association, the International Society for Telemedicine and Telehealth, WHO e-health, and the International Telehealth and Telemedicine Accreditation URAC.

Finally, an internal expert panel revised the indicators and then suggested additional indicators based on the panel’s experience in the field of quality and telemedicine. A long list of indicators was developed and sorted into domains and subdomains that were guided by telehealth accreditation standards called URAC accreditation standards [27].

### Phase 3, modified Delphi method

A modified Delphi method was employed with a panel of experts. The purpose of applying the Delphi method was to reach an agreement on a set of KPIs based on the chosen criteria. The Delphi was administered in the period from December 2022 to May 2023. In this study, two rounds were needed for the anticipated level of consensus.

#### Recruitment of the experts

Purposive sampling was employed to ensure the selection of proper experts. These experts were recruited through personal contacts as well as through a contact list provided by AVH. The sample included telemedicine experts, healthcare providers in telemedicine services, healthcare quality experts, and public health experts. They were selected based on these criteria: having 5 years in the field of healthcare services or quality; being employed in the hospital field; having knowledge or experience in performance indicators; having knowledge or experience in telemedicine; and being interested in participating in the study. An invitation e-mail was sent to each expert, outlining the study’s aim, explaining the Delphi method, and including an informed consent.

A pilot study was conducted with three panelists before the first round to refine the Delphi questionnaire. In this stage, the questionnaire items and format were finalized. The pilot study results were not included in the final Delphi results.

#### Round one in Delphi

Round one was done through an online questionnaire for the indicators. The indicators were presented with the domains and subdomains which were related to the URAC accreditation standards. In each domain, an open-ended question was added for any other KPIs, as suggested by the experts. Each indicator was presented to the participants with a clear definition (as shown in the supplementary materials). The experts were requested to assign a rating (on a 10-point Likert scale ranging from 1 for “strongly disagree” to 10 for “strongly agree”) to each indicator based on the three selection criteria. This 10-point scale facilitated a clear distribution of opinions. After a month, the response was collected, and the data were analyzed, making sure that they were anonymously handled. A standard descriptive statistical analysis for the first round included: the percentage of agreement, the mean, the standard deviation (SD), and the median and interquartile range (IQR) to be presented in round 2 [28–30].

#### Round two in Delphi

As a clear definition of each indicator was presented already to the participants, the selection criterion “clearly defined” was removed in the second round based on the agreement of all experts involved in this study. The statistical results from the first round were sent to the experts for the second round of the Delphi technique. Each expert had an individual sheet presenting his/her own rating with the descriptive data of experts’ opinion from the first round (mean, SD, median, IQR of the data group). The experts were requested to re-evaluate the indicators based on the feedback received. This enabled participants to adjust their responses in consideration of the group’s opinions. The re-rating was determined by only two selection criteria (i.e. feasibility and importance) following the same 10-rating scale. The questionnaire for the second round was the same as for the first one but included 6 additional indicators suggested by experts in the first round. To ensure consistency in the study, only the experts who took part in the first round of the Delphi method were invited to participate in the second round.

#### The final list of indicators

After finishing the Delphi technique, the final list of indicators determined whether or not more than or equal to 70% of responders agreed on the importance and feasibility of the indicator. Kappa statistics for the indicators were calculated to compare the results from the first and second rounds, indicating the percentage of agreement between them.

### Data management and analysis

The collected data were reviewed, coded, and organized into tables before being entered into a PC using the Statistical Package for the Social Sciences (SPSS 20 for Windows). Data cleaning, quality checks, and data entry were carried out. The data were presented, and appropriate analyses were conducted based on the type of data obtained for each parameter. The quantitative analysis from the Delphi method included calculating: the response rates, the percentages for each level of agreement for each indicator, as well as the median, interquartile range (IQR), mean, standard deviation (SD), and the Cohen Weighted Kappa (K).

Weighted Kappa (K) statistics were computed to assess the level of agreement regarding the importance and feasibility rankings within subjects between the two rounds, rather than the level of agreement among participants [31, 32]. To elaborate, Weighted Kappa is an extension of Cohen’s Kappa, which is widely employed for measuring agreement [33, 34]. The Kappa values were utilized to compare the agreement between rounds for each statement in the Delphi method, illustrating the stability of responses, as noted in various studies [35–39].

## Results

### Selection criteria

After reviewing studies, a long list of selection criteria that would be used in the Delphi questionnaires was revealed. The long list included more than 40 criteria that were refined to eliminate redundancy and repeated criteria, and to design a shortened list of 17 selection criteria. A multi-voting system was used among a panel of experts to select the top 3 criteria. The panel came up with the following selection criteria: clearly defined (i.e. the unambiguity of the indicator), the feasibility (i.e. the possibility to collect the required data with reasonable efforts), and the importance (i.e. the impact on health/service, or the measurement capturing something that makes a difference in the service effectiveness).

### A potential list of indicators

The review of sources was conducted to explore indicators to start the Delphi from credible sources as WHO, Pan American Health Organization, National Quality Forum and studies which focused on telemedicine evaluation. Out of 191 studies from research sources reviewed, none of them focused on the indicators that directly related to performance measurement in telemedicine. Most of the studies centered on evaluating telemedicine through the customer perceptions of the service, and on comparisons between telemedicine use and in-person healthcare services. Another significant portion of the studies addressed the measurement of clinical outcomes for telemedicine patients. These types of measures were not designed to assess the performance of a telemedicine setting from a quality measurement perspective. Thus, this highlights a clear gap in knowledge within this area of study.

Throughout the internal expert panel, a long potential list of 60 indicators was identified after reviewing the studies. These indicators were: utilization, satisfaction, and technical measures. After research for the meanings of some indicators was done, it was revealed that some of them were ambiguous, replicated, or irrelevant to telemedicine services. Consequently, the final long list included 52 indicators. The list was categorized into three main domains: operations, clinical services, and customer satisfaction. Each main domain was categorized into subdomains.

### Participants in the modified Delphi technique

**Fig. 1:**
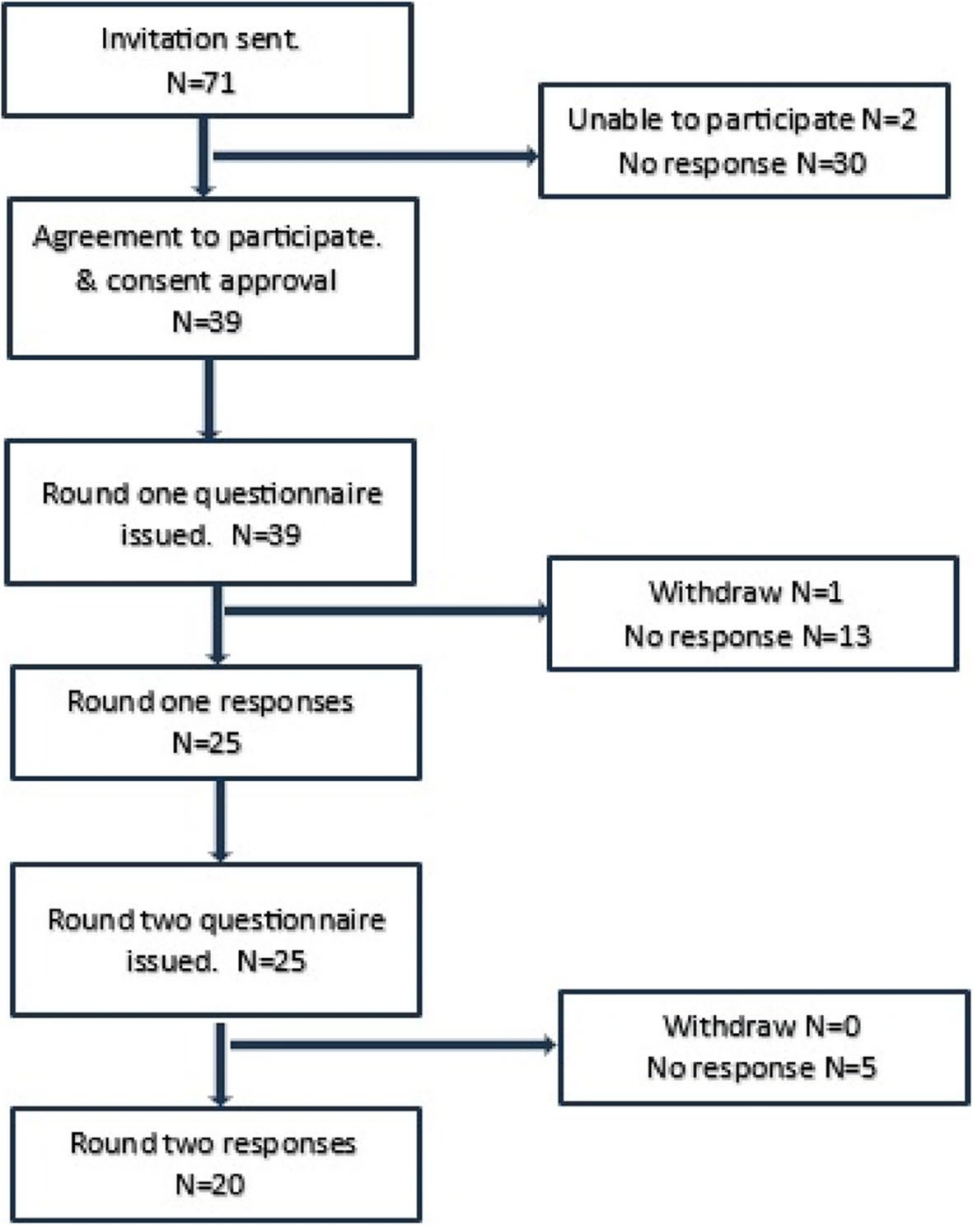
Flow of participants through the Delphi method

The Delphi method process is illustrated in (Fig. 1). Out of the 71 experts invited, 25 panelists participated in the first round, and of those, 20 experts took part in the second round. The response rates for the two rounds were 35% and 80%, respectively as shown in (Table 1).

**Table 1:** Response rate of the expert in Delphi

Expert Categories	Invited	Response Rate % in first round	Response Rate % in the second round
Quality Experts	13	9 (69%)	9 (100%)
Telemedicine Experts^*^	5	4 (80%)	3 (75%)
Public Health Experts	11	7 (63%)	7 (100)
Healthcare Providers in AVH	42	5 (12%)	1 (20%)
**Total**	**71**	**25 (35%)**	**20 (80%)**

^*^Three of the telemedicine experts are the virtual hospital management team

Most experts who participated in the previous round also engaged in the subsequent round. The qualifications of the experts involved in the Delphi process are shown in (Table 2).

**Table 2:** Qualifications of experts involved in the Delphi rounds

Items	Categories	First Round		Second Round	
		Number	Percentage (%)	Number	Percentage (%)
**Education Background**	PhD	22	88%	17	85%
	Master’s Degree	2	8%	2	10%
	Bachelor’s Degree	1	4%	1	5%
**Workplace**	Hospital	16	64%	12	60%
	Academic Institution	8	32%	8	32%
	Health Agency	1	4%	0	0%
**Quality Experience**	Yes	9	36%	9	45%
	No	17	68%	11	55%
**Telemedicine Experience**	Yes	9	36%	5	25%
	No	17	68%	15	75%
**Total**		25		20	

### Round one and two Delphi

**Fig. 2:**
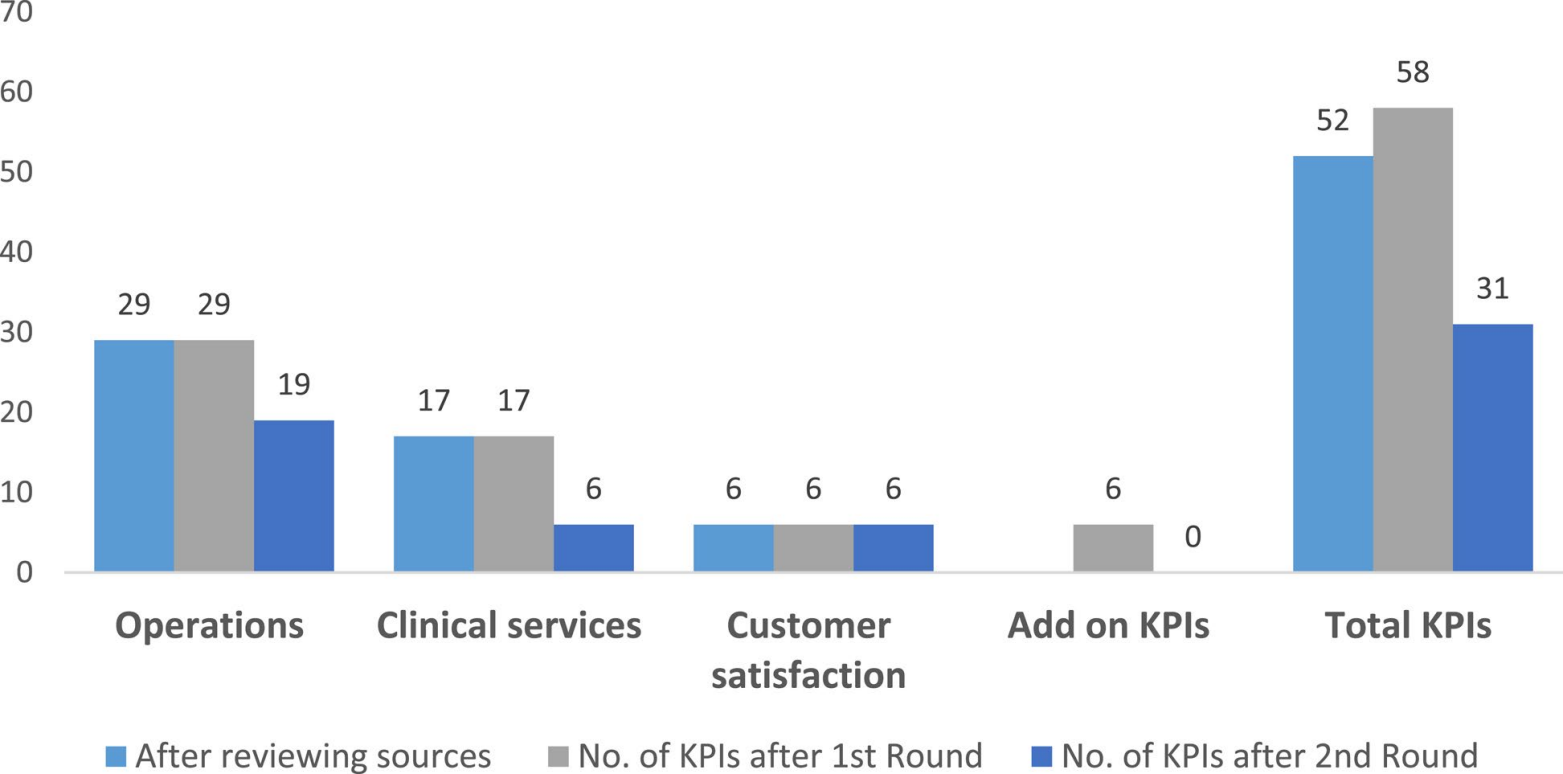
Numbers of KPIs in different phases in the study

**Fig. 3:**
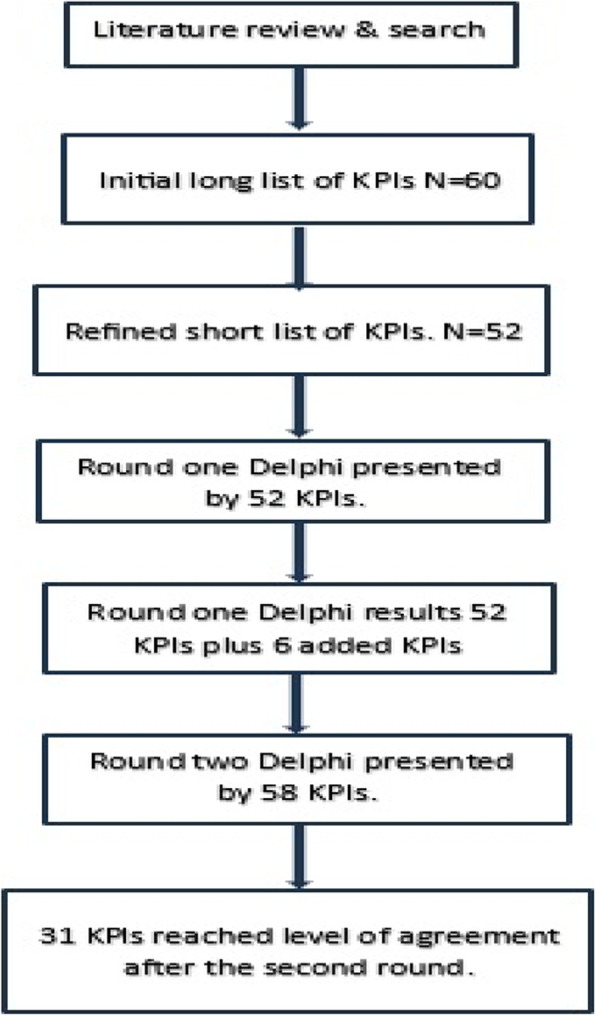
Flowchart of KPIs through the study

The results from the first and second rounds were analyzed using SPSS. After the first round, there were 52 KPIs and 6 more which were suggested by the experts. The second round which was sent to each expert included 58 KPIs. The results of the Delphi method are as follows: of the 58 KPIs, 31 KPIs reached the 70% level of agreement in feasibility and importance criteria (Table 3). The 6 add-on KPIs did not reach the level of agreement in the second round. As a result, almost 60% of indicators reached the level of agreement (Fig. 2). The flowchart of KPIs through the study was demonstrated in (Fig. 3).

**Table 3:** Indicators which reached the 70% level of agreement after round 2

Domain	Subdomain	Selected Indicators	Mean & SD for feasibility	Mean & SD for importance
Operations	Access	1) Average waiting time to access service	8.95 ± 1.605	9.00 ± 1.451
		2) Average waiting to receive the service	8.60 ± 1.603	8.80 ± 1.196
		3) Average Consultation time (contact time)	8.55 ± 1.820	7.55 ± 1.932
	Training	4) Percentage of staff oriented on using the video consultation	8.55 ± 1.356	8.30 ± 1.559
		5) Percentage of cases educated on technical support upon request	8.20 ± 1.576	7.50, 1.792
	Utilization	6) Total number of video consultation visits per month	9.25 ± 1.070	8.55 ± 1.432
		7) Proportion of video consultations done with cases from outside Egypt	8.85 ± 1.309	7.50 ± 1.638
		8) Utilization rate	8.70 ± 1.342	8.45 ± 1.538
		9) Percentage of no-show cases	8.85 ± 1.981	8.20 ± 2.419
		10) Percentage of no-show providers	9.00 ± 1.747	9.10 ± 1.774
		11) Cancellation rate for Video-consultation	9.25 ± 1.070	9.45 ± 1.276
	System management	12) Server uptime	8.25 ± 1.970	8.20 ± 1.609
		13) Number of Server unplanned down time	8.70 ± 1.780	8.85 ± 1.927
		14) Percentage of Server Availability	7.35 ± 2.159	7.80 ± 2.016
		15) Percentage of errors in the pre-call testing	8.60 ± 1.536	9.05 ± 1.099
		16) Number of system disasters per 3 months	8.30 ± 2.055	8.25 ± 1.997
		17) Duration of Implementation solution in response to disaster	7.75 ± 2.149	8.35 ± 2.033
	Information Security	18) Duration of preventive measures implementation after security incident	7.65 ± 2.110	7.85 ± 2.134
	Supplier Management	19) Number of identified contract breaches annually	7.95 ± 1.669	7.45 ± 1.731
Clinical services	Patient Demographics	20) Patient variation (according to age, gender, diagnosis)	9.35 ± 0.933	8.10 ± 1.447
	Clinical Indicators	21) Percentage of clinically deteriorated diabetic patients with follow up visits	7.45 ± 1.638	7.70 ± 1.418
	Referral/transition of care	22) Percentage of patient transfer to a hospital (hospitalization)	8.15 ± 1.755	8.15 ± 2.084
		23) Percentage of patients transferred to ER.	8.00 ± 1.947	8.15 ± 2.300
		24) Percentage of patient referral to other specialty	8.10 ± 1.997	7.75 ± 1.860
		25) Percentage of patient referral to onsite consultation (face to face)	7.85 ± 2.033	8.55 ± 1.572
Customer satisfaction		26) Percentage of patient satisfaction	8.90 ± 1.410	9.20 ± 1.005
		27) Net Promoter Score	8.70 ± 1.218	9.20 ± 0.951
		28) Percentage of addressed patient complaints	7.85 ± 2.059	8.70 ± 1.342
		29) Annual provider’s turnover rate	8.35 ± 2.390	8.00 ± 2.317
		30) Percentage of staff satisfaction	9.10 ± 1.210	9.25 ± 0.851
		31) Percentage of addressed staff complaints	8.05 ± 2.481	9.10 ± 1.373

The Weighted Kappa values for each indicator, indicating the degree of agreement between the first and second rounds, ranged from 0.32 to 0.96, with a mean of 0.7325. The Kappa values were interpreted based on previous studies [35] as follows: 0.0–0.2 indicated poor agreement, 0.21–0.4 indicated fair agreement, 0.41–0.6 indicated moderate agreement, 0.61–0.8 indicated substantial agreement, and 0.81–1 indicated almost perfect agreement. Nearly 85% of the comparisons between the two rounds in this study fell within the substantial agreement range and were statistically significant (< 0.001), while approximately 36% of the comparisons were within the almost perfect agreement range and were also statistically significant (< 0.001). For that reason, these values confirm the reliability of the consensus process. The selected indicators cover a broad range of quality dimensions (Table 4).

**Table 4:** Relevant dimension of quality and type for each indicator

Selected Indicators	Relevant Dimension of Quality	Type of indicators
**Operations**		
1) Average waiting time to access service	Access & Timeliness	Process
2) Average waiting to receive the service		
3) Average Consultation time (contact time)		
4) Percentage of staff oriented on using the video consultation	Competency	Process
5) Percentage of cases educated on technical support upon request	Patient Centeredness	Process
6) Total number of video consultation visits per month	Access & Efficiency	Outcome
7) Proportion of video consultations done with cases from outside Egypt	Access & Equity	Outcome
8) Utilization rate	Efficiency	Outcome
9) Percentage of no-show cases	Access & Efficiency	Outcome
10) Percentage of no-show providers	Timeliness & Efficiency	Outcome
11) Cancellation rate for Video-consultation	Efficiency	Outcome
12) Server uptime	Efficiency	process
13) Number of Server unplanned down time	Efficiency	Outcome
14) Percentage of Server Availability	Access	Process
15) Percentage of errors in the pre-call testing	Effectiveness	Outcome
16) Number of system disasters per 3 months		
17) Duration of Implementation solution in response to disaster	Timeliness	Process
18) Duration of preventive measures implementation after security incident		
19) Number of identified contract breaches annually	Accountability & Effectiveness	Process
**Clinical Services**		
20) Patient variation (according to age, gender, diagnosis)	Patient Centeredness & Equity	Outcome
21) Percentage of clinically deteriorated diabetic patients with follow up visits	Effectiveness	Outcome
22) Percentage of patient transfer to a hospital (hospitalization)	Continuity	Process
23) Percentage of patients transferred to ER.		
24) Percentage of patient referral to other specialty		
25) Percentage of patient referral to onsite consultation (face to face)		
**Customer Satisfaction**		
26) Percentage of patient satisfaction	Patient centered	Outcome
27) Net Promoter Score		
28) Percentage of addressed patient complaints		
29) Annual provider’s turnover rate	Efficiency	Structure
30) Percentage of staff satisfaction	Staff Engagement & Efficiency	Outcome
31) Percentage of addressed staff complaints		

## Discussion

The study addresses a significant and underexplored area—performance measurement in telemedicine, particularly in a low- and middle-income countries. A review of the scientific literature revealed a notable gap, with few indicators explicitly designed for telemedicine performance evaluation. This gap underscores the need for a structured approach to identify and validate relevant indicators.

To address this gap, the Delphi technique was employed as it represents a well-established methodology for reaching a consensus of expert opinions on a specific topic [40–43]. To elaborate further, Delphi helps researchers to clarify problems; develop questionnaire statements for rating; select panelists to rate them; and conduct feedback between rounds. Numerous studies have employed this approach to determine a set of indicators [44–47].

In the current study, the first round in Delphi yielded a low response rate of 35% despite sending reminders and offering flexible timelines to the panel. This limited engagement, particularly from healthcare providers, likely reflects the challenges that these professionals face (such as demanding workloads, time constraints, limited availability due to their clinical responsibilities, and their low knowledge of the importance of KPIs). The lower response rate may be due to the larger number of items included in the Delphi. As some studies show, the larger the items, the lower the response rate [48, 49]. Additionally, the low response rate from the invited panel may exacerbate the limitations associated with the unclear methodological design inherent in the Delphi method [50, 51].

The selected panel included professionals from different disciplines, such as quality experts, telemedicine experts as the AVH management team, healthcare providers in AVH, and public health experts. However, future studies could further benefit from incorporating additional strategies, such as involving other important stakeholders as patients and technical staff; providing incentives; employing more follow-up measures to encourage greater participation; and/or educating the stakeholders on how KPIs benefit their work and patient outcomes.

The chosen domains were developed in alignment with international accreditation standards, encompassing operations, clinical services, and customer satisfaction [27]. The operations domain characterized the integrity of the hospital’s system, which was further divided into access, training, utilization trends, system management and efficiency, information security, and supplier management. Generally, these domains align with international best practices while addressing unique contextual needs in Egypt, such as system efficiency and equitable access to telemedicine services. In this study, there were two subdomains that had the large number of KPIs: utilization trends and system management; both are very important for telemedicine performance.

The utilization indicators provide insights into the effectiveness and efficiency of services provided [52, 53]. From the 9 KPIs in utilization subdomain, 6 KPIs were selected. These KPIs help in understanding how resources are being utilized within the system and in taking corrective actions to improve the quality and efficiency of the service provided. In addition, the KPIs help in forecasting future demand and planning capacity [54, 55]. The number of teleconsultations was measured in many studies [56–58]. In addition, some other studies measured the number of no-show patients as well as the total cancellations [57, 59].

While there are studies on telemedicine that often focus on patient satisfaction and clinical outcomes, this particular study expands the scope by incorporating operational and system management metrics. In the system management subdomain, 6 KPIs were selected from 11 KPIs. Previous studies focused on measuring technical difficulties or errors in teleconsultation as important measures of utilization [56, 60, 61]. The current study suggested many measures beyond technical difficulties, such as server uptime, unplanned downtime, server availability, and system disaster, highlighting the importance of IT reliability in telemedicine. Undoubtedly, system management indicators are crucial for maintaining the reliability, performance, and availability of IT systems; in other words, they are essential for supporting business operations and achieving organizational goals.

As mentioned in the report of National Quality Forum [62], access to care domain was vital for the evaluation of telemedicine services. In addition, this framework supported the idea of standardizing the evaluation and focused on the main measures, such as the waiting times. Analyzing patient wait times is crucial for healthcare settings (including telemedicine) to understand and manage the situation as it may result in patient dissatisfaction and may negatively affect healthcare outcomes. In this study, all 3 proposed KPIs within the access subdomain were retained in both rounds, indicating the significance of measuring waiting times, as mentioned in previous studies [57, 59, 63–65]. From the 3 proposed KPIs for training, 2 indicators were selected, reflecting the orientation of the new staff on using the video consultation and continuous patient education on technical support upon request.

The clinical service`s domain focused on patients’ stations during their care and the different services provided in the hospital. The indicators from this domain were primarily outcome measures. Of the 17 indicators, only 6 indicators were selected: patient demographics, percentage of clinically deteriorated diabetic patients with follow-up visits, transfer of patients to admission in a hospital, ER, other specialty, or face-to-face consultation.

The patient demographics section aims to provide insights into the characteristics of the patient population being served to tailor services to meet their specific needs and identify disparities in healthcare access and outcomes [57, 61]. As diabetes mellitus is a common diagnosis, the selected indicator to measure the percentage of clinically-deteriorated diabetic patients with follow-up visits plays a crucial role in evaluating healthcare quality, disease management effectiveness, prevention of complications, and patient engagement in the care process of the disease [66, 67].

The selection of clinical outcome indicators was limited, with a narrow focus on patient-centered KPIs that address direct health outcomes, such as improved health metrics or safety indicators. This limitation arose as the AVH platform’s system has not yet sufficiently developed to support remote patient monitoring, as it only includes video consultation sessions and medical records. This constraint was acknowledged at the beginning of the study. Therefore, it is recommended that future studies focus more on patient-centered outcomes as they are crucial for addressing the aspects of care that matter the most to patients, including their health status and quality of life.

Transition of care indicators are metrics that are used to assess the quality and effectiveness of transitions in healthcare, particularly as patients move between different healthcare settings or stages of care. These indicators help ensure the continuity of care and positive outcomes [68, 69]. In the present study, all the 4 proposed KPIs of transition of care were selected. Previous studies also demonstrated the transition of care indicators [63, 70–73] as Ferguson et al. measured the transfer rate from video consultations to teaching hospitals [70]. These indicators align with the frameworks that emphasize the continuity of care [11].

All the 6 indicators of customer satisfaction were selected. This domain is very important in measuring the performance of any organization, and the indicators are some of the patient-centered outcomes. Generally, the customers are either internal ones, such as the hospital staff, or external ones, such as the patients. Measuring their satisfaction with the service provided is crucial; this can identify strengths, weaknesses, and opportunities for improvement, leading to enhanced customer experiences, increased loyalty, and improved business performance. These indicators highlight its significance, as demonstrated in various prior studies before and after COVID-19 [64, 74–78]. Patient satisfaction with the service is a key patient-centered outcome. These outcomes help ensure that healthcare delivery is aligned with patients’ preferences, needs, and values, fostering more effective and individualized care.

The developed list of KPIs offers a vital tool for telemedicine providers, policymakers and quality improvement teams. By systematically measuring and evaluating access, utilization and satisfaction, these measures can guide data-driven decision making, enhance accountability, and improve delivery of services in telemedicine settings. The framework also supports the alignment of telemedicine services with Egypt’s goals for the Universal Health Coverage, guaranteeing equitable and high-quality care for all stakeholders.

### Study strengths

The selection of the Delphi method was based on various factors; one being the diversity of expertise across different fields and environments. Moreover, the Delphi technique involved iterative rounds of expert opinion ratings, allowing experts to include or exclude indicators as necessary. Another benefit was the absence of face-to-face interactions among panelists as it minimized biases -such as dominance by vocal participants- and encouraged independent thinking by eliminating group pressure [79].

The range of experts included in this study reflected those providing telemedicine services. Invitations were extended to all medical practitioners and managerial personnel at AVH, as well as senior quality staff at Ain Shams University hospitals. This broad inclusion contributed to the authenticity of the study, surpassing the limitations of solely relying on KPIs derived from existing literature [80, 81].

### Study limitations

The response rate for the first round of the modified Delphi was relatively low, mainly due to the large pool of potential experts identified, and due to the voluntary nature of participation in the study. Given that the main sector of the experts consisted of healthcare providers, the response rate was notably low. This could be potentially attributed to the experts’ limited familiarity with the indicators and their significance in assessing organizational performance. The low response rate may have implications for the representativeness of the results, as a broader input from providers would ensure that the selected KPIs are both practical and relevant to telemedicine settings.

Furthermore, there might be a selection bias in the panel of experts as this study depended on the experts’ (i.e. quality experts, public health experts) familiarity with the KPIs. To ensure a more comprehensive perspective in future panels, it is recommended to include a broader range of stakeholders (such as patients, IT specialists, and policymakers) in future studies. This diverse inclusion would provide a well-rounded view, incorporating various viewpoints and expertise, and would reduce the risk of bias.

Additionally, the study focuses solely on AVH as a telemedicine setting, limiting the generalizability of findings to other telemedicine contexts. It is suggested to adapt and validate these KPIs in other hospitals or regions to enhance generalizability.

## Conclusion

Enhancing the quality of a virtual hospital necessitates staff-establishing performance indicators pertinent to service areas. In this study, consensus was reached on 31 Key Performance Indicators (KPIs) for monitoring performance at Ain Shams Virtual Hospital (AVH). The study offers significant contributions to the field by laying the groundwork for consensus on such type of KPIs. Telemedicine Organizations should consider integrating these KPIs into their standard practices to enhance performance evaluation.

The selected KPIs should be applied and measured in real-world settings to assess their practicality, relevance, and effectiveness in evaluating telemedicine services. This approach will help determine if the KPIs accurately reflect performance and will lead to meaningful improvements in patient care and operational efficiency.

## Supplementary Information

Supplementary Material 1.

## Data Availability

The authors confirms that all data generated or analyzed during this study are included in this manuscript and supplementary information files.

## Abbreviations

KPIs: Key Performance Indicators
AVH: Ain Shams Virtual Hospital
WHO: World Health Organization
URAC: Utilization Review Accreditation Commission
K: Cohen Kappa
SD: Standard Deviation
IQR: Interquartile Range
PC: Personal Computer
SPSS: Statistical Package for the Social Sciences
ER: Emergency Room
IT system: Information Technology
