# Supplementary material for: Development of key performance indicators for a telemedicine setting in Egypt using an electronic modified Delphi approach
Source: BMC Health Serv Res. 2025 Jul 1;25:868. doi: 10.1186/s12913-025-12733-6 (PMC12220657; doi:10.1186/s12913-025-12733-6)
Supplement: Supplementary file 1 — Supplementary Material 1. [file 12913_2025_12733_MOESM1_ESM.zip › Round one Delphi questionnaire.docx]

**Round 1 – Delphi**

**Consensus on a set of key performance indicators**

To take this questionnaire, please open it from laptop or rotate the phone to see all the options.

You can edit your response to complete the questionnaire in another sessions.

For each Key Performance Indicator, please rate the level of agreement according to each criteria (from your point of view) - from 1 to 10 ( ***1 strongly disagree and 10 strongly agree***). Please remember that there are no right or wrong answers to the questions, only this technique is seeking your expert opinion.

**Definitions of the selection criteria**

- Clearly defined: the degree to which the indicator is unambiguous.
- Feasibility: the possibility to collect the required data with reasonable efforts.
- Importance or impact on health/ service: the measurement captures something that make a difference in the service effectiveness.

| **KPIs** | **Clearly defined** | **Feasibility** | **Importance / impact on health or service** |
| --- | --- | --- | --- |
| 1. **Operations** | | | |
| **Access (waiting time)** | | | |
| 1. Average waiting time to access service   (Definition: time to first appointment availability) |  |  |  |
| 1. Average waiting to receive the service   (Def. time when the patient enters the lobby till fetching the patient by the provider – will be stratified by specialty and by provider) |  |  |  |
| 1. Average Consultation time / contact time   (Def. time from the first fetching of the patient by the provider to the time of the session ended – will be stratified by specialty and by provider) |  |  |  |
| **Training** | | | |
| 1. Percentage of staff oriented on using the video consultation )technical support( |  |  |  |
| 1. Percentage of educated patients on technical support before the usage of the system |  |  |  |
| 1. Percentage of patients educated on technical support upon request |  |  |  |
| **Utilization trends:** | | | |
| 1. Total number of video consultation visits per month   (will be stratified by Customer – Provider and Provider -Provider) |  |  |  |
| 1. Proportion of video consultations done with patients from outside Egypt |  |  |  |
| 1. Utilization rate   (Numerator: number of operated hours in the system – denominator: number of available hours in the system – it will be stratified by specialty and provider) |  |  |  |
| 1. Percentage of no-show patients |  |  |  |
| 1. Percentage of no-show providers |  |  |  |
| 1. Cancellation rate for Video-consultation   (will be stratified by providers, specialty, and cause) |  |  |  |
| 1. Number of App downloads quarterly |  |  |  |
| 1. Hospitalization rate (within 30 days) after using the virtual hospital) |  |  |  |
| 1. Percentage of postoperative video consultation visits   (by using the treats platform???) |  |  |  |
| **System management/ business efficiency/ performance:** | | | |
| 1. Percentage of Server Availability   (Def. availability is the percentage of time, in a specific time interval, during which a server can be used for its intended purpose, Availability % = Uptime/Total time, Total time = Uptime + Downtime,  Downtime: an unexpected shutdown or failure of the server). |  |  |  |
| 1. Server uptime   (Uptime refers to the time a system runs without a shutdown or restart. Server uptime is the total duration for which a server is fully functional and running) |  |  |  |
| 1. Number of server unplanned down time   (Def. an unexpected shutdown or failure of the server) |  |  |  |
| 1. Percentage of errors related to browsers: ……………………..   (Def. technical issues related to the type of the browser e.g chrome, Firefox …… The errors will be stratified by the type of browser and if it happened with the providers or the patients  Numerator: number of technical errors related to the browser, denominator: number of times using the same browser) |  |  |  |
| 1. Percentage of errors in the precall testing   (Precall testing: a test occurs in the precall phase to check the mic, camera and connections… The errors will be stratified by the type of errors  Numerator: number of errors in the precall testing (technical errors that was predefined in the system)  Denominator: the number of precall testing occurred) |  |  |  |
| 1. Video visit successful completion rate (Success rate) |  |  |  |
| 1. Hiccup rate   (Def. video or audio difficulty that can be overcome either by changing the platforms or brief troubleshooting e.g refreshing the browser and the session completed,  Numerator: number of hiccups, Denominator: number of all sessions) |  |  |  |
| 1. Number of service interruptions per month   (in case of patient or provider exit before completing the session, camera failure, mic failure, network failure - these causes whether the session will be completed or not) |  |  |  |
| 1. Average duration of service interruptions per month   (will be stratified by specialty) |  |  |  |
| 1. Number of system disasters per 3 months   (System failure or breakdown or personal errors plus system failure) |  |  |  |
| 1. Duration of Implementation solution in response to disaster   (Def. duration from the identification of a disaster-related risk to the implementation of a suitable continuity mechanism) |  |  |  |
| **Information security:** | | | |
| 1. No. of major security incidents.   (Def. security incident is an event that may indicate that an organization's systems or data have been compromised or that measures put in place to protect them have failed.) |  |  |  |
| 1. Duration of preventive measures implementation after security incident   (Def. Duration from the identification of a security threat to the implementation of a suitable counter measure) |  |  |  |
| **Supplier management:** |  |  |  |
| 1. Number of identified contract breaches annually   (Def. number of contractual obligations which were not fulfilled by suppliers (identified during contract reviews) |  |  |  |
| 1. **Clinical service** | | | |
| **Patient demographics:** | | | |
| 1. Patient variation (according to age, gender, diagnosis) |  |  |  |
| **Biometrics:** | | | |
| 1. Percentage of diabetic patients with no complications in the first visit   (Numerator: number of diabetic patients with no complications in the first visit, denominator: number of all diabetic patients) |  |  |  |
| 1. Percentage of clinically deteriorated diabetic patients with follow up visits   (Numerator: number of diabetic patients developed complications in follow up visits, denominator: all diabetic patients) |  |  |  |
| 1. Percentage of diabetic patients aged 20 years or more with three or more A1C tests per year   )Numerator: number of diabetic patients with 3 or more A1C per year, denominator: number of all diabetic patients in the follow up visits) |  |  |  |
| 1. Percentage of diabetic patients aged 20 years or more with micro albuminuria - a positive urine screening test is an indicator of early kidney damage.   (Numerator: number of diabetic patients with positive urine screening, denominator: number of all diabetic patients) |  |  |  |
| 1. The percentage of diabetic patients aged 20 years or more with a most recent A1C level less than or equal to 7% as the optimal target for glycemic control.   (Numerator: number of diabetic patients with A1C less or equal to 7% in the follow up visits, denominator: number of all diabetic patients) |  |  |  |
| 1. Percentage of diabetic patients 18-75 years old who had A1C > 9% during the measurement period.   (Numerator: number of diabetic patients with A1C more than 9% in the follow up visits, denominator: number of all diabetic patients) |  |  |  |
| 1. Percentage of HTN patients 18-85 years of age with the most recent blood pressure was adequately controlled (<140/90mmhg) during the measurement period.   (Numerator: number of HTN patients with controlled blood pressure in the follow up visits, denominator: number of all HTN patients) |  |  |  |
| Please add Others (if any)…… |  |  |  |
| **Medication errors:** | | | |
| 1. Number of not to be used abbreviations   (Not to be used abbreviations: U/IU, Q.D, QD, q.o.d, qod, MS, MSO4,MgSO4, No trailing zero, no leading zero) |  |  |  |
| 1. Number of Adverse drug reactions reported by patients   (in the Treats platform) |  |  |  |
| **Antibiotic prescription:** | | | |
| 1. Percent of AB prescription per 100 patients.   (for most common, most expensive and most serious antibiotics) |  |  |  |
| **Symptom scale & screening process :** | | | |
| 1. Percentage of patients screened for depression   (Numerator: number of patients screened for depression, denominator: number of target group of patients) |  |  |  |
| 1. Percentage of patient with positive screening test result for “depression” in the follow up visits   (Numerator: number of patients with positive screening test for depression in the follow up visits, denominator: number of all screened patients). |  |  |  |
| Please add Others (if any)…… |  |  |  |
| **Referral/ transition of care:** | | | |
| 1. Percentage of patient transfer to a hospital (hospitalization) |  |  |  |
| 1. Percentage of patients transferred to ER. |  |  |  |
| 1. Percentage of patient referral to other specialty |  |  |  |
| 1. Percentage of patient referral to onsite consultation (face to face) |  |  |  |
| **C)Customer satisfaction** | | | |
| 1. Percentage of patient satisfaction   (measured by patient satisfaction survey after the video consultation) |  |  |  |
| 1. Net Promoter Score   (Overall customer perception or loyalty) |  |  |  |
| 1. Percentage of addressed patient complaints.   (numerator: The number of patient complaints that have been dealt with, Denominator: total number of patient`s complaints per month) |  |  |  |
| 1. Annual providers turnover rate   (Def. the number of employees who quit the organization, or, are asked to leave, and are replaced by the new employees. Calculated by No. of providers who left/((No. at the beginning of the year+ No. at the end of the year)/2)*100) |  |  |  |
| 1. Percentage of staff satisfaction |  |  |  |
| 1. Percentage of addressed staff complaints   (numerator: The number of staff complaints that have been dealt with, denominator: total number of staff complaints per month) |  |  |  |
