# Supplementary material for: Development of key performance indicators for a telemedicine setting in Egypt using an electronic modified Delphi approach
Source: BMC Health Serv Res. 2025 Jul 1;25:868. doi: 10.1186/s12913-025-12733-6 (PMC12220657; doi:10.1186/s12913-025-12733-6)
Supplement: Supplementary file 1 — Supplementary Material 1. [file 12913_2025_12733_MOESM1_ESM.zip › Supplementary materials.docx]

**Long List of criteria after literature review**

| 1. Clearly defined. | 1. Accepted Practice and History of Use |
| --- | --- |
| 1. Impact on health | 1. Applicability in Different Settings |
| 1. Susceptibility to being influenced by healthcare system. | 1. Availability of Data |
| 1. Availability of data across the system | 1. Burden of Data Collection on Participants |
| 1. Cost of collecting data | 1. Clarity of Focus and Meaning |
| 1. Acceptability | 1. Cultural Appropriateness and Relevance |
| 1. Relevance | 1. Data Quality |
| 1. Accuracy | 1. Investment of Resources |
| 1. Importance | 1. Nondirectional Language |
| 1. Usefulness | 1. Opportunity to Detect Unexpected or Unintended Findings |
| 1. Feasibility | 1. Pathway for Use of Data |
| 1. Credibility | 1. Relevance to Evaluation Questions |
| 1. Validity | 1. Strength of Evidence or Substantive Merit |
| 1. Distinctiveness | 1. Value within a Set of Indicators |
| 1. to the needs of the user | 1. Valid |
| 1. based on available or easily accessible data | 1. easily communicable |
| 1. performance-based | 1. Transparent |
| 1. Broadly accepted. | 1. Simple |
| 1. Easy to interpret. | 1. Accessible data |
| 1. Quantifiable | 1. Sensitive |
| 1. Meaningful/ Easily understood. | 1. Performance-based |
| 1. Timely | 1. User-friendly |
| 1. Standardized | 1. Precision |
| 47- Specific | 1. Capacity to upscale |
| 1. Affordable measurement | |

**A table showed a short list of the selection criteria:**

| 3- Measurable | 2- Non-directional Language | 1. Clearly defined /simple   (Clarity of Meaning) |
| --- | --- | --- |
| 6- Measure High Volume process | 5- Accuracy / Validity | 4- Reliable |
| 9- Related to Patient Safety Goals | 8- Measure Problem prone service | 7- Measure High-cost service |
| 12- Importance or impact on health | 11- Acceptability | 10- Relevant with AVH mission and/ or specialty |
| 15- Opportunity to Detect Unexpected or Unintended Findings | 14- Credibility | 13- Feasibility |
| 17- Value within a Set of Indicators | | 16- Strength of Evidence or Substantive Merit |

**The 52 indicators after literature review:**

| 1. **Operations** |
| --- |
| **Access (waiting time)** |
| 1. Average waiting time to access service. |
| 1. Average waiting to receive the service. |
| 1. Average Consultation time / contact time |
| **Training** |
| 1. Percentage of staff oriented on using the video consultation (technical support( |
| 1. Percentage of patients watched the videos of technical support before using the system |
| 1. Percentage of patients educated on technical support upon request |
| **Utilization trends:** |
| 1. Total number of video consultation visits per month |
| 1. Proportion of video consultations done with patients from outside Egypt |
| 1. Utilization rate |
| 1. Percentage of no-show patients |
| 1. Percentage of no-show providers |
| 1. Cancellation rate for Video-consultation |
| 1. Number of App downloads quarterly |
| 1. Hospitalization rate (within 30 days) after using the virtual hospital) |
| 1. Percentage of postoperative video consultation visits |
| **System management/ business efficiency/ performance:** |
| 1. Percentage of Server Availability |
| 1. Server uptime |
| 1. Number of server unplanned down time |
| 1. Percentage of errors related to browsers |
| 1. Percentage of errors in the pre-call testing |
| 1. Video visit successful completion rate (Success rate) |
| 1. Hiccup rate |
| 1. Number of service interruptions per month |
| 1. Average duration of service interruptions per month |
| 1. Number of system disasters per 3 months |
| 1. Duration of Implementation solution in response to disaster |
| **Information security:** |
| 1. No. of major security incidents. |
| 1. Duration of preventive measures implementation after security incident |
| **Supplier management:** |
| 1. Number of identified contract breaches annually |
| 1. **Clinical service** |
| **Patient demographics:** |
| 1. Patient variation (according to age, gender, diagnosis) |
| **Biometrics:** |
| 1. Percentage of diabetic patients with no complications in the first visit |
| 1. Percentage of clinically deteriorated diabetic patients with follow up visits |
| 1. Percentage of diabetic patients aged 20 years or more with three or more A1C tests per year |
| 1. Percentage of diabetic patients aged 20 years or more with micro albuminuria - a positive urine screening test is an indicator of early kidney damage. |
| 1. The percentage of diabetic patients aged 20 years or more with a most recent A1C level less than or equal to 7% as the optimal target for glycemic control. |
| 1. The percentage of diabetic patients 18-75 years old who had A1C > 9% during the measurement period. |
| 1. Percentage of HTN patients 18-85 years of age with the most recent blood pressure was adequately controlled (<140/90mmhg) during the measurement period. |
| Please add Others (if any)…… |
| **Medication errors:** |
| 1. Number of not to be used abbreviations |
| 1. Number of Adverse drug reactions reported by patients |
| **Antibiotic prescription:** |
| 1. Percent of AB prescription per 100 patients. |
| **Symptom scale & screening process :** |
| 1. Percentage of patients screened for depression |
| 1. Percentage of patient with positive screening test result for “depression” in the follow up visits |
| Please add Others (if any)…… |
| **Referral/ transition of care:** |
| 1. Percentage of patient transfer to a hospital (hospitalization) |
| 1. Percentage of patients transferred to ER. |
| 1. Percentage of patient referral to other specialty |
| 1. Percentage of patient referral to onsite consultation (face to face) |
| **C)Customer satisfaction** |
| 1. Percentage of patient satisfaction |
| 1. Net Promoter Score |
| 1. Percentage of addressed patient complaints. |
| 1. Annual providers turnover rate |
| 1. Percentage of staff satisfaction |
| 1. Percentage of addressed staff complaints |

**Weighted Kappa Coefficient values between the two rounds for each criterion.**

| Ratings | Weighted Kappa | Std Error | Z | Significance | 95% LCL | 95% UCL |
| --- | --- | --- | --- | --- | --- | --- |
| v1_Feas.round_1 - v1_Feas.round_2 | 0.88 | 0.05 | 4.32 | <0.001 | 0.79 | 0.97 |
| v1_impor.round_1 - v1_impor.round_2 | 0.91 | 0.05 | 4.26 | <0.001 | 0.81 | 1.00 |
| v2_Feas.round_1 - v2_Feas.round_2 | 0.74 | 0.20 | 3.57 | <0.001 | 0.35 | 1.12 |
| v2_impor.round_1 - v2_impor.round_2 | 0.72 | 0.12 | 3.71 | <0.001 | 0.48 | 0.96 |
| v3_Feas.round_1 - v3_Feas.round_2 | 0.93 | 0.04 | 4.23 | <0.001 | 0.85 | 1.01 |
| v3_impor.round_1 - v3_impor.round_2 | 0.60 | 0.16 | 2.91 | <0.001 | 0.28 | 0.92 |
| v4_Feas.round_1 - v4_Feas.round_2 | 0.83 | 0.10 | 3.79 | <0.001 | 0.63 | 1.04 |
| v4_impor.round_1 - v4_impor.round_2 | 0.84 | 0.11 | 3.95 | <0.001 | 0.62 | 1.05 |
| v5_Feas.round_1 - v5_Feas.round_2 | 0.64 | 0.19 | 2.97 | <0.001 | 0.26 | 1.02 |
| v5_impor.round_1 - v5_impor.round_2 | 0.71 | 0.11 | 3.28 | <0.001 | 0.50 | 0.91 |
| v6_Feas.round_1 - v6_Feas.round_2 | 0.84 | 0.11 | 3.80 | <0.001 | 0.62 | 1.06 |
| v6_impor.round_1 - v6_impor.round_2 | 0.79 | 0.10 | 3.58 | <0.001 | 0.59 | 0.99 |
| v7_Feas.round_1 - v7_Feas.round_2 | 0.85 | 0.10 | 4.06 | <0.001 | 0.66 | 1.04 |
| v7_impor.round_1 - v7_impor.round_2 | 0.63 | 0.13 | 3.42 | <0.001 | 0.39 | 0.88 |
| v8_Feas.round_1 - v8_Feas.round_2 | 0.84 | 0.08 | 4.00 | <0.001 | 0.69 | 0.99 |
| v8_impor.round_1 - v8_impor.round_2 | 0.58 | 0.17 | 3.02 | <0.001 | 0.25 | 0.91 |
| v9_Feas.round_1 - v9_Feas.round_2 | 0.79 | 0.12 | 3.78 | <0.001 | 0.56 | 1.02 |
| v9_impor.round_1 - v9_impor.round_2 | 0.72 | 0.12 | 3.82 | <0.001 | 0.49 | 0.95 |
| v10_Feas.round_1 - v10_Feas.round_2 | 0.84 | 0.10 | 3.97 | <0.001 | 0.65 | 1.04 |
| v10_impor.round_1 - v10_impor.round_2 | 0.86 | 0.06 | 3.89 | <0.001 | 0.75 | 0.97 |
| v11_Feas.round_1 - v11_Feas.round_2 | 0.90 | 0.06 | 4.21 | <0.001 | 0.78 | 1.01 |
| v11_impor.round_1 - v11_impor.round_2 | 0.87 | 0.06 | 3.98 | <0.001 | 0.75 | 0.99 |
| v12_Feas.round_1 - v12_Feas.round_2 | 0.54 | 0.14 | 3.38 | <0.001 | 0.27 | 0.81 |
| v12_impor.round_1 - v12_impor.round_2 | 0.56 | 0.16 | 3.56 | <0.001 | 0.24 | 0.88 |
| v13_Feas.round_1 - v13_Feas.round_2 | 0.68 | 0.22 | 3.21 | <0.001 | 0.25 | 1.10 |
| v13_impor.round_1 - v13_impor.round_2 | 0.32 | 0.22 | 1.50 | 0.13 | -0.11 | 0.75 |
| v14_Feas.round_1 - v14_Feas.round_2 | 0.93 | 0.03 | 4.19 | <0.001 | 0.86 | 1.00 |
| v14_impor.round_1 - v14_impor.round_2 | 0.70 | 0.19 | 3.29 | <0.001 | 0.32 | 1.08 |
| v15_Feas.round_1 - v15_Feas.round_2 | 0.86 | 0.08 | 3.91 | <0.001 | 0.71 | 1.02 |
| v15_impor.round_1 - v15_impor.round_2 | 0.55 | 0.21 | 2.58 | <0.001 | 0.14 | 0.97 |
| v16_Feas.round_1 - v16_Feas.round_2 | 0.79 | 0.14 | 3.82 | <0.001 | 0.52 | 1.07 |
| v16_impor.round_1 - v16_impor.round_2 | 0.64 | 0.17 | 3.29 | <0.001 | 0.31 | 0.98 |
| v17_Feas.round_1 - v17_Feas.round_2 | 0.65 | 0.16 | 3.27 | <0.001 | 0.34 | 0.96 |
| v17_impor.round_1 - v17_impor.round_2 | 0.59 | 0.18 | 2.74 | 0.01 | 0.24 | 0.95 |
| v18_Feas.round_1 - v18_Feas.round_2 | 0.72 | 0.13 | 3.56 | <0.001 | 0.46 | 0.97 |
| v18_impor.round_1 - v18_impor.round_2 | 0.84 | 0.07 | 3.86 | <0.001 | 0.70 | 0.98 |
| v19_Feas.round_1 - v19_Feas.round_2 | 0.76 | 0.12 | 3.61 | <0.001 | 0.51 | 1.00 |
| v19_impor.round_1 - v19_impor.round_2 | 0.58 | 0.13 | 2.78 | 0.01 | 0.32 | 0.83 |
| v20_Feas.round_1 - v20_Feas.round_2 | 0.80 | 0.08 | 3.77 | <0.001 | 0.65 | 0.96 |
| v20_impor.round_1 - v20_impor.round_2 | 0.82 | 0.11 | 3.72 | <0.001 | 0.61 | 1.03 |
| v21_Feas.round_1 - v21_Feas.round_2 | 0.77 | 0.09 | 3.63 | <0.001 | 0.59 | 0.95 |
| v21_impor.round_1 - v21_impor.round_2 | 0.66 | 0.15 | 3.12 | <0.001 | 0.36 | 0.96 |
| v22_Feas.round_1 - v22_Feas.round_2 | 0.77 | 0.11 | 3.60 | <0.001 | 0.57 | 0.98 |
| v22_impor.round_1 - v22_impor.round_2 | 0.66 | 0.11 | 3.40 | <0.001 | 0.45 | 0.88 |
| v23_Feas.round_1 - v23_Feas.round_2 | 0.70 | 0.13 | 3.40 | <0.001 | 0.45 | 0.95 |
| v23_impor.round_1 - v23_impor.round_2 | 0.82 | 0.08 | 3.88 | <0.001 | 0.67 | 0.98 |
| v24_Feas.round_1 - v24_Feas.round_2 | 0.64 | 0.14 | 3.08 | <0.001 | 0.36 | 0.93 |
| v24_impor.round_1 - v24_impor.round_2 | 0.75 | 0.11 | 3.54 | <0.001 | 0.52 | 0.97 |
| v25_Feas.round_1 - v25_Feas.round_2 | 0.76 | 0.13 | 3.61 | <0.001 | 0.51 | 1.02 |
| v25_impor.round_1 - v25_impor.round_2 | 0.54 | 0.20 | 2.53 | 0.01 | 0.15 | 0.93 |
| v26_Feas.round_1 - v26_Feas.round_2 | 0.79 | 0.09 | 3.71 | <0.001 | 0.62 | 0.97 |
| v26_impor.round_1 - v26_impor.round_2 | 0.71 | 0.11 | 3.53 | <0.001 | 0.50 | 0.92 |
| v27_Feas.round_1 - v27_Feas.round_2 | 0.84 | 0.06 | 3.80 | <0.001 | 0.72 | 0.95 |
| v27_impor.round_1 - v27_impor.round_2 | 0.78 | 0.10 | 3.57 | <0.001 | 0.60 | 0.97 |
| v28_Feas.round_1 - v28_Feas.round_2 | 0.84 | 0.07 | 3.83 | <0.001 | 0.70 | 0.99 |
| v28_impor.round_1 - v28_impor.round_2 | 0.75 | 0.11 | 3.42 | <0.001 | 0.53 | 0.96 |
| v29_Feas.round_1 - v29_Feas.round_2 | 0.81 | 0.11 | 3.72 | <0.001 | 0.58 | 1.03 |
| v29_impor.round_1 - v29_impor.round_2 | 0.48 | 0.28 | 2.18 | 0.03 | -0.07 | 1.02 |
| v30_Feas.round_1 - v30_Feas.round_2 | 0.70 | 0.14 | 3.20 | <0.001 | 0.42 | 0.97 |
| v30_impor.round_1 - v30_impor.round_2 | 0.53 | 0.20 | 2.52 | 0.01 | 0.14 | 0.93 |
| v31_Feas.round_1 - v31_Feas.round_2 | 0.88 | 0.06 | 4.03 | <0.001 | 0.76 | 0.99 |
| v31_impor.round_1 - v31_impor.round_2 | 0.67 | 0.10 | 3.38 | <0.001 | 0.48 | 0.86 |
| v32_Feas.round_1 - v32_Feas.round_2 | 0.86 | 0.08 | 3.91 | <0.001 | 0.70 | 1.01 |
| v32_impor.round_1 - v32_impor.round_2 | 0.47 | 0.19 | 2.31 | 0.02 | 0.11 | 0.84 |
| v33_Feas.round_1 - v33_Feas.round_2 | 0.57 | 0.22 | 2.59 | 0.01 | 0.13 | 1.01 |
| v33_impor.round_1 - v33_impor.round_2 | 0.74 | 0.14 | 3.47 | <0.001 | 0.47 | 1.02 |
| v34_Feas.round_1 - v34_Feas.round_2 | 0.79 | 0.17 | 3.59 | <0.001 | 0.46 | 1.11 |
| v34_impor.round_1 - v34_impor.round_2 | 0.43 | 0.21 | 2.11 | 0.03 | 0.02 | 0.84 |
| v35_Feas.round_1 - v35_Feas.round_2 | 0.50 | 0.22 | 2.33 | 0.02 | 0.08 | 0.93 |
| v35_impor.round_1 - v35_impor.round_2 | 0.54 | 0.17 | 2.43 | 0.02 | 0.20 | 0.88 |
| v36_Feas.round_1 - v36_Feas.round_2 | 0.66 | 0.20 | 3.03 | <0.001 | 0.26 | 1.06 |
| v36_impor.round_1 - v36_impor.round_2 | 0.43 | 0.19 | 2.00 | 0.05 | 0.06 | 0.80 |
| v37_Feas.round_1 - v37_Feas.round_2 | 0.57 | 0.22 | 2.60 | 0.01 | 0.14 | 0.99 |
| v37_impor.round_1 - v37_impor.round_2 | 0.48 | 0.18 | 2.21 | 0.03 | 0.13 | 0.83 |
| v38_Feas.round_1 - v38_Feas.round_2 | 0.59 | 0.22 | 2.81 | 0.01 | 0.16 | 1.02 |
| v38_impor.round_1 - v38_impor.round_2 | 0.67 | 0.15 | 3.07 | <0.001 | 0.37 | 0.97 |
| v39_Feas.round_1 - v39_Feas.round_2 | 0.46 | 0.23 | 2.14 | 0.03 | 0.02 | 0.90 |
| v39_impor.round_1 - v39_impor.round_2 | 0.68 | 0.16 | 3.08 | <0.001 | 0.37 | 0.99 |
| v40_Feas.round_1 - v40_Feas.round_2 | 0.80 | 0.12 | 3.63 | <0.001 | 0.56 | 1.03 |
| v40_impor.round_1 - v40_impor.round_2 | 0.75 | 0.15 | 3.40 | <0.001 | 0.45 | 1.05 |
| v41_Feas.round_1 - v41_Feas.round_2 | 0.96 | 0.03 | 4.31 | <0.001 | 0.92 | 1.01 |
| v41_impor.round_1 - v41_impor.round_2 | 0.61 | 0.15 | 2.72 | 0.01 | 0.31 | 0.91 |
| v42_Feas.round_1 - v42_Feas.round_2 | 0.77 | 0.13 | 3.54 | <0.001 | 0.52 | 1.03 |
| v42_impor.round_1 - v42_impor.round_2 | 0.68 | 0.14 | 3.22 | <0.001 | 0.41 | 0.96 |
| v43_Feas.round_1 - v43_Feas.round_2 | 0.95 | 0.03 | 4.28 | <0.001 | 0.89 | 1.01 |
| v43_impor.round_1 - v43_impor.round_2 | 0.91 | 0.02 | 4.10 | <0.001 | 0.87 | 0.96 |
| v44_Feas.round_1 - v44_Feas.round_2 | 0.89 | 0.03 | 4.21 | <0.001 | 0.82 | 0.96 |
| v44_impor.round_1 - v44_impor.round_2 | 0.87 | 0.05 | 3.91 | <0.001 | 0.77 | 0.96 |
| v45_Feas.round_1 - v45_Feas.round_2 | 0.90 | 0.04 | 3.94 | <0.001 | 0.83 | 0.97 |
| v45_impor.round_1 - v45_impor.round_2 | 0.81 | 0.10 | 3.57 | <0.001 | 0.62 | 1.01 |
| v46_Feas.round_1 - v46_Feas.round_2 | 0.92 | 0.05 | 4.16 | <0.001 | 0.83 | 1.01 |
| v46_impor.round_1 - v46_impor.round_2 | 0.85 | 0.06 | 3.82 | <0.001 | 0.74 | 0.96 |
| v47_Feas.round_1 - v47_Feas.round_2 | 0.96 | 0.02 | 4.34 | <0.001 | 0.92 | 1.01 |
| v47_impor.round_1 - v47_impor.round_2 | 0.81 | 0.10 | 3.94 | <0.001 | 0.61 | 1.00 |
| v48_Feas.round_1 - v48_Feas.round_2 | 0.40 | 0.25 | 2.05 | 0.04 | -0.09 | 0.88 |
| v48_impor.round_1 - v48_impor.round_2 | 0.59 | 0.13 | 3.38 | <0.001 | 0.33 | 0.85 |
| v49_Feas.round_1 - v49_Feas.round_2 | 0.89 | 0.07 | 4.02 | <0.001 | 0.76 | 1.01 |
| v49_impor.round_1 - v49_impor.round_2 | 0.86 | 0.08 | 3.89 | <0.001 | 0.71 | 1.01 |
| v50_Feas.round_1 - v50_Feas.round_2 | 0.91 | 0.06 | 4.11 | <0.001 | 0.80 | 1.02 |
| v50_impor.round_1 - v50_impor.round_2 | 0.79 | 0.13 | 3.59 | <0.001 | 0.54 | 1.04 |
| v51_Feas.round_1 - v51_Feas.round_2 | 0.85 | 0.10 | 3.97 | <0.001 | 0.67 | 1.04 |
| v51_impor.round_1 - v51_impor.round_2 | 0.94 | 0.04 | 4.22 | <0.001 | 0.86 | 1.01 |
| v52_Feas.round_1 - v52_Feas.round_2 | 0.79 | 0.12 | 3.62 | <0.001 | 0.56 | 1.03 |
| v52_impor.round_1 - v52_impor.round_2 | 0.79 | 0.12 | 3.67 | <0.001 | 0.56 | 1.03 |
